# Discovery of cancer-preventive juices reactivating RB functions

**DOI:** 10.1265/ehpm.23-00160

**Published:** 2023-09-22

**Authors:** Mitsuharu Masuda, Mano Horinaka, Shusuke Yasuda, Mie Morita, Emi Nishimoto, Hideki Ishikawa, Michihiro Mutoh, Toshiyuki Sakai

**Affiliations:** 1 Department of Molecular-Targeting Prevention, Kyoto Prefectural University of Medicine; 2 Department of Drug Discovery Medicine, Kyoto Prefectural University of Medicine

**Keywords:** Retinoblastoma gene, Cancer prevention, Kakadu plum, Pomegranate, Juice, Lactic acid bacteria, Tumor necrosis factor-related apoptosis-inducing ligand, Anti-inflammatory effects, Antioxidant effects

## Abstract

**Background:**

Recent advances have been achieved in the genetic diagnosis and therapies against malignancies due to a better understanding of the molecular mechanisms underlying carcinogenesis. Since active preventive methods are currently insufficient, the further development of appropriate preventive strategies is desired.

**Methods:**

We searched for drinks that reactivate the functions of tumor-suppressor retinoblastoma gene (RB) products and exert anti-inflammatory and antioxidant effects. We also examined whether lactic acid bacteria increased the production of the cancer-specific anti-tumor cytokine, tumor necrosis factor-related apoptosis-inducing ligand (TRAIL), in human, and examined whether the RB-reactivating drinks with lactic acid bacteria decreased azoxymethane-induced rat colon aberrant crypt foci (ACF) and aberrant crypts (ACs) *in vivo*.

**Results:**

Kakadu plum juice and pomegranate juice reactivated RB functions, which inhibited the growth of human colon cancer LIM1215 cells by G1 phase arrest. These juices also exerted anti-inflammatory and antioxidant effects. *Lactiplantibacillus* (*L.) pentosus* S-PT84 was administered to human volunteers and increased the production of TRAIL. In an *in vivo* study, Kakadu plum juice with or without pomegranate juice and S-PT84 significantly decreased azoxymethane-induced rat colon ACF and ACs.

**Conclusions:**

RB is one of the most important molecules suppressing carcinogenesis, and to the best of our knowledge, this is the first study to demonstrate that natural drinks reactivated the functions of RB. As expected, Kakadu plum juice and pomegranate juice suppressed the growth of LIM1215 cells by reactivating the functions of RB, and Kakadu plum juice with or without pomegranate juice and S-PT84 inhibited rat colon ACF and ACs. Therefore, this mixed juice has potential as a novel candidate for cancer prevention.

**Supplementary information:**

The online version contains supplementary material available at https://doi.org/10.1265/ehpm.23-00160.

## Background

The development of preventive strategies, diagnostic modalities, and therapies is crucial for reducing the mortality rates of malignant tumors. Due to advances in our understanding of the molecular mechanisms underlying carcinogenesis, improvements have been achieved in molecular diagnoses and therapies. Since the screening of germ-line mutations for the diagnosis of a predisposition to cancer is now rapid and easy, ‘preemptive medicine’, which requires preventive agents for each malignant tumor, is considered to be very important. However, very few molecular preventive agents are currently available because research on preventive interventions is time consuming and expensive, making companies hesitate to develop them. Nevertheless, we and others have shown that cyclooxygenase-2 (COX-2) inhibitors, such as aspirin and celecoxib, are promising agents for the prevention of human colorectal tumors [1–4]. Due to severe adverse events, such as bleeding in the duodenum, stomach, or brain, these treatments are not recommended to everyone. The majority of drugs are associated with adverse effects and, thus, are not suitable for long-term administration to healthy individuals. Based on previous findings showing that fruits prevent various malignant tumors [5, 6], the use of fruit juice with cancer-preventive effects may be an alternative preventive strategy against cancer due to its safety. Another advantage of fruit juice is that it may be easily combined with other effective juices and lactic acid bacteria and so on.

The retinoblastoma gene (RB) was discovered as the first tumor suppressor gene [7]. It was subsequently shown to be inactivated at the protein level in the majority of malignant tumors [8], and, thus, is regarded as the most important molecule suppressing carcinogenesis at the so-called R-point in G1 phase of the cell cycle distinguishing normal and malignant cells [9, 10]. We previously demonstrated that many cancer-preventive or growth inhibitory food factors, such as vitamin D3, sulforaphane, flavone, butyrate, apigenin, all-trans retinoic acid, brassinin, indole-3-carbinol, sesamin, sesaminol, fucoxanthin, artepillin C, cryptolepine, vitamin K2, dehydrozingerone, perillyl alcohol, arctiin, L-canavanine, and resibufogenin, activated the functions of RB, suggesting the importance of RB for cancer prevention [11–32].

We also reported that the quantification of RB functions was useful for the diagnosis of gastrointestinal cancers [33], and this system has been used as a diagnostic service for the prognosis of early breast cancer [34]. Furthermore, we proposed a novel screening method that detects agents enhancing RB protein activity to pharmaceutical companies. We then discovered the first-in-class and best-in-class MEK inhibitor trametinib (trade name, Mekinist) [35] against BRAF mutant malignancies, such as melanoma, non-small cell lung cancer, anaplastic thyroid cancer, and other 20 malignancies [8]. Trametinib suppressed tumorigenesis in a mouse model of familial adenomatous polyposis and early-stage sporadic colon cancer formation by decreasing COX-2 levels [36]. Therefore, agents that reactivate RB functions have potential as very good candidates for cancer therapy and prevention [8].

Another important cancer preventive molecule is the cancer-specific anti-tumor cytokine, tumor necrosis factor-related apoptosis-inducing ligand (TRAIL). TRAIL specifically induces apoptosis in only cancer cells that express TRAIL receptors. Previous studies showed that this effect of TRAIL was highly specific to cancer cells, and it is expected to exert cancer-preventive effects because a TRAIL deficiency was found to promote malignancies in mice [37]. The genus *Lactiplantibacillus* is one of the most common indigenous lactic acid bacteria in humans, and is widely used in the production of fermented foods. We already demonstrated that the addition of plant-derived lactic acid bacteria (three strains of *L. plantarum* S1, DB22, and DS41) to peripheral blood mononuclear cells (PBMC) promoted the expression of TRAIL at the mRNA and protein levels and facilitated natural killer (NK) activity against cancer cells [38]. In the present study, we confirmed that a different species of the genus *Lactiplantibacillus* exerted TRAIL-inducing effects in a human volunteer study.

We performed a screening of 25 drinks in a world, including fruit juices, vegetable juices, teas, and so on, reactivating functions of the RB protein. We then found that Kakadu plum (*Terminalia ferdinandiana*) juice and pomegranate (*Punica granatum*) juice reactivated the RB functions, which is first to show that regular drinks can activate the functions of RB. Whereas pomegranate is cultivated in many countries including Japan, Kakadu plum is a traditional bush food of the Aboriginal people in Australia and is also used as a cosmetic ingredient.

Other than RB and TRAIL, anti-inflammatory and antioxidant effects are considered to be important for cancer prevention, and the results obtained herein confirmed that Kakadu plum juice and pomegranate juice both exerted these effects. Furthermore, Kakadu plum juice with or without pomegranate juice and S-PT84 inhibited rat colon aberrant crypt foci (ACF) and aberrant crypts (ACs) *in vivo*, suggesting that the mixed juice has potential in the prevention of cancer.

## Methods

### Cells, chemicals, and reagents

Human colon carcinoma LIM1215 cells from a hereditary non-polyposis colon cancer patient were purchased from CellBank Australia [39]. LIM1215 cells have a hetero missense mutation of CTNNB1/β-catenin, whereas other important genes for cell growth regulation, such as APC, p53, PIK3CA, BRAF, and KRAS, are wild-type, which is similar to the early stage of colon cancer [39]. Kakadu plum juice was obtained from Sanyo Foods Co., Ltd. (Tokyo, Japan). Pomegranate juice was supplied by Oyama Co., Ltd. (Kobe, Japan). From-concentrate juices with pasteurization were obtained from each supplier. Each juice was filtered through 0.45 µm-filter to remove insoluble components, then aliquoted and stored at −80 °C until assay. Corilagin was purchased from Cayman Chemical Co. (Ann Arbor, MI, USA) and punicalagin from Sigma-Aldrich (St. Louis, MO, USA).

Rabbit anti-phospho-RB (Ser807/811) (#9308) and rabbit anti-phospho-RB (Ser780) (#9307) were obtained from Cell Signaling Technology (Danvers, MA, USA). Mouse anti-RB (#554136) was purchased from BD Pharmingen (San Diego, CA, USA), mouse anti-glyceraldehyde-3-phosphate dehydrogenase (GAPDH) from HyTest (Turku, Finland), rabbit polyclonal anti-TRAIL (sc-56243) and rabbit anti-p21 (sc-397) from Santa Cruz Biotechnology (Dallas, TX, USA), and horseradish peroxidase (HRP)-conjugated anti-rabbit IgG (#NA934V) and anti-mouse IgG (#NA931V) from GE Healthcare/Cytiva. Immobilon western chemiluminescent HRP substrate was obtained from Millipore (Temecula, CA, USA), Dulbecco’s modified Eagle’s medium (DMEM) from Nissui (Tokyo, Japan), and the High-Capacity cDNA Reverse Transcription Kit from Applied Biosystem (Waltham, MA, USA). Chemi-Lumi One L, phosphatase inhibitor cocktail, and Sepasol-RNA I Super G were supplied by Nacalai Tesque (Kyoto, Japan). Azoxymethane (AOM) was purchased from Sigma-Aldrich, medetomidine hydrochloride, butorphanol tartrate, and tumor necrosis factor-α (TNF-α) from Fuji Film Wako Pure Chemical Corporation (Yokohama, Japan), and midazolam from Fuji Pharma (Tokyo, Japan). The primers/probes for interleukin 6 (IL-6) (Hs00985639_m1) and GAPDH (Hs99999905_m1) in the TaqMan Assay were obtained from Thermo Fisher Scientific (Waltham, MA, USA). CM-H_2_DCFDA was supplied by Invitrogen (Waltham, MA, USA). Ficoll-Paque PLUS was purchased from GE Healthcare/Cytiva (Tokyo, Japan).

### Cell passage culture

LIM1215 cells were routinely cultured in DMEM supplemented with 10% fetal bovine serum, 50 units/mL penicillin, 100 µg/mL streptomycin, and 4 mM L-glutamine.

### Colony formation assay

LIM1215 cells were seeded on 6-well plates at a density of 300 cells per well. On the next day, cells were treated with several volumes of each juice. As a control, cells were treated with PBS. The total volume of juice with PBS added to each well was fixed at 50 µL. The pH of culture media was maintained at neutral by the addition of sodium hydrogen carbonate. After an incubation for an additional 2 weeks, cells were fixed with 4% neutral buffered formalin and then stained with 0.1% crystal violet. The number of stained colonies was visually counted.

### Flow cytometric analysis of the cell cycle distribution

LIM1215 cells were seeded on 6-well plates (1 × 10^5^ cells in 2 mL medium/well). On the next day, medium was changed to non-serum medium for the synchronization of the cell cycle and cells were cultured for an additional 2 days. Medium was then replaced with fresh medium containing 10% fetal bovine serum and the respective concentrations of juice. As a control, cells were treated with PBS instead of juice. After the incubation of cells for 24 hours with or without each juice, cells were harvested by a trypsin treatment and centrifuged at 1,500 rpm at 4 °C for 5 minutes. Cells were suspended in PBS containing 0.2% Triton X-100 and 50 µg/mL propidium iodide. Stained cells were analyzed using FACSCalibur (Becton, Dickinson and Company, Franklin Lakes, NJ, USA). Data were analyzed using Cell Quest software (Becton, Dickinson and Company, Franklin Lakes, NJ, USA) and ModFit LT software (Verity Software House, Topsham, ME, USA) for the cell cycle.

### Western blotting analysis

Cells were incubated with the selected juices for 72 hours. As a control, cells were incubated with PBS. After washing with PBS, cells were lysed in lysis buffer containing 50 mM Tris-HCl (pH 7.5), 1.0% SDS, 1 mM DTT, 0.43 mM 4-(2-aminoethyl) benzenesulfonyl fluoride hydrochloride, and phosphatase inhibitor cocktail. Lysates were sonicated and centrifuged, and the supernatant was collected for sodium dodecyl sulfate-polyacrylamide gel electrophoresis (SDS-PAGE). An equal amount of the protein extract was subjected to SDS-PAGE and transferred to a polyvinylidene difluoride membrane.

The membrane was treated with a blocking buffer, washed, and then reacted with the primary antibody for each protein at room temperature for 1 hour or at 4 °C overnight. After washing, the secondary antibody was reacted at room temperature for 1 hour. The signals of protein bands were detected by reacting with a luminescent reagent and exposure to an X-ray film.

### Anti-inflammatory assay

Cells were seeded on 12-well plates (5 × 10^5^ cells in 1 mL medium/well). After 2 days, cells were cultured with each juice for 1 hour. Inflammation events were induced by 10 ng/mL TNF-α. As a control, cells were treated with PBS instead of juice. After an incubation with TNF-α for 1 hour, total RNA was isolated from cells using Sepasol-RNA I Super G. Total RNA (2–10 µg/tube) was reversely transcribed to cDNA in a 10-µL reaction volume using the High-Capacity cDNA Reverse Transcription Kit. An equivalent volume of cDNA solution was used for quantitative RT-PCR. cDNA was amplified and quantified using TaqMan probes for IL-6 and GAPDH. The quantity of IL-6 mRNA was normalized by GAPDH mRNA.

### Flow cytometric analysis to measure intracellular reactive oxygen species (ROS)

Cells were seeded on 12-well plates (4 × 10^4^ cells in 1 mL medium/well). On the next day, cells were treated with each juice for 24 hours. As a control, cells were treated with PBS. Cells were treated with 500 µM hydrogen peroxide (H_2_O_2_) to induce ROS for the last 1 hour of the 24-hour juice treatment. After the incubation, cells were washed with PBS and incubated for 30 minutes with 10 µM CM-H_2_DCFDA. CM-H_2_DCFDA is oxidized by ROS in cells and finally becomes 2′, 7′-dichlorodihydrofluorescein (DCF), which emits strong fluorescence. Cells were collected by trypsin treatment and centrifuged at 1,500 rpm, 4 °C for 5 minutes. Cells were suspended in PBS and the fluorescence of DCF was measured using FACSCalibur to determine the amount of intracellular ROS. We analyzed the data using the Cell Quest software.

### Preparation of freeze-dried lactic acid bacteria

*Lactiplantibacillus* (*L.*) *pentosus* S-PT84 was isolated from Kyoto pickles, cultured in de Man, Rogosa, and Sharpe broth (Difco Laboratories, Detroit, MI, USA) at 37 °C for 24 hours, and harvested via centrifugation. Bacteria were washed twice with sterile saline, washed again with distilled water, and heat-killed at 95 °C for 5 minutes. No colonies formed on agar plates, confirming the non-viability of heat-killed S-PT84. Heat-killed S-PT84 was freeze-dried and used in subsequent experiments.

### Human study design and subjects

Twenty-one healthy volunteers (23–47 years) were adopted as subjects. Subjects were recruited at the Kyoto Prefectural University of Medicine and were divided into an experimental group and a control group. They took a placebo tablet (starch tablet, n = 5) or S-PT84 tablet (n = 16) (0.5 billion of S-PT84 (n = 5), 1.5 billion of S-PT84 (n = 6) or 4.5 billion of S-PT84 (n = 5)) in the morning every day for 4 weeks. Blood samples were collected 4 times: 1 week before the ingestion of tablets, 2 weeks and 4 weeks after the start of intake, and 4 weeks after the period of intake. PBMC were isolated from whole blood using Ficoll-Paque PLUS according to the manufacturer’s instructions. Western blotting was performed as described above. Rabbit polyclonal anti-TRAIL and mouse monoclonal anti-GAPDH antibodies were used as primary antibodies. The intensity of the TRAIL band was quantified using ImageJ and corrected by the expression level of GAPDH.

The study protocol was approved by the Ethics Committee of Kyoto Prefectural University of Medicine (RBMR-C-1010). Written informed consent was obtained from all subjects. We registered this study to UMIN-CTR (University Hospital Medical Information Network Clinical Trials Registry). The registered ID is UMIN000007114.

### Animals

Five-week-old male F344 rats were purchased from Charles River (Wilmington, MA, USA). After one week of acclimation, animals were randomly grouped into control or experimental groups. All animal studies were approved by the Animal Care and Use Committee of Kyoto Prefectural University of Medicine (approval number M28-569). The CRF-1 pellet diet was purchased from Oriental Yeast Co., Ltd. (Tokyo, Japan).

### Model of AOM-induced rat colon ACF

Rats were quarantined for one week before being randomized into two groups. One group was orally administered a 1.5 mL sample on five consecutive days each week for 4 weeks. The other group was treated with water on the same schedule. All rats had free access to the CRF-1 pellet diet and water during the experimental periods. All rats were subcutaneously administered AOM (15 mg/kg body weight) on days 2 and 8.

Kakadu plum juice was administered in the first experiment. In the second experiment, a mixed juice of 50% Kakadu plum juice and 50% pomegranate juice with 0.01 g of dry powdered *L. pentosus* S-PT84 (5 × 10^10^ CFU/1 g of dry powder) was administered. At the end of the study (week 5), all rats were euthanized with anesthesia (0.03 mg/mL medetomidine hydrochloride, 0.5 mg/mL butorphanol tartrate, and 0.4 mg/mL midazolam) and colonic lesions were removed. The colon was flushed with saline, cut open longitudinally along the main axis, washed with saline, and then fixed in 10% neutral buffered formalin. ACF and ACs stained with 0.2% methylene blue were counted under a microscope (×10–40 magnification).

### Statistical analysis

Statistical analyses of the results of the anti-inflammatory assay on each juice were conducted by Dunnett’s multiple comparison test. The inhibition rate of colony formation, a cell cycle analysis, qRT-PCR of IL-6, and intracellular ROS measurements were examined by Tukey’s multiple comparison procedure. In the human study, the significance of differences between groups was performed by an unpaired *t*-test with Welch’s correction. Differences in the numbers of ACF or ACs between the control group and experimental group were examined by the Student’s *t*-test.

## Results

### Inhibitory effects of fruit juices on cell growth

Fruit juices generally contain many acidic compounds, such as vitamin C and organic acids, and are strongly acidic. These conditions may affect cell growth by lowering the pH of the culture medium. Therefore, before evaluating the effects of fruit juice on colony formation, we examined the impact of pH on colony formation in a preliminary study. Regarding colony formation in culture media adjusted to various pH by an inorganic acid (HCl) or organic acid (citrate), a marked decrease was observed in the colony formation rate when pH was less than approximately 7.0 (data not shown). In the present study, the pH of the medium with each juice sample ranged between approximately 7.0 and 8.0 without the direct effects of pH on cell growth (data not shown).

**Fig. 1:**
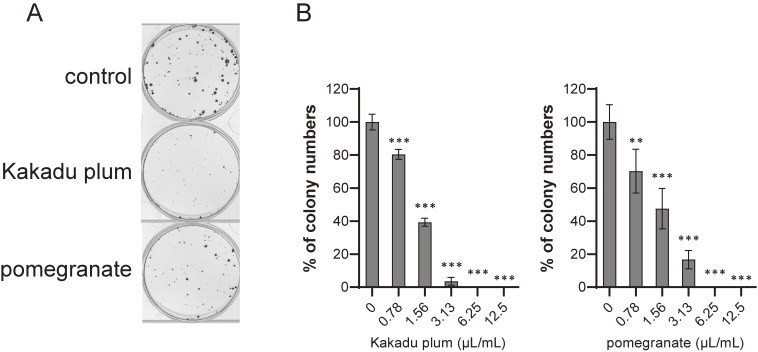
Effects of Kakadu plum juice and pomegranate juice on colony formation by LIM1215 cells. A. Clonogenic assay of LIM1215 cells treated with each juice (1.56 µL/mL) for 2 weeks. B. Cells were cultured with each concentration of each juice for 2 weeks. Data represent the means ± SD of three measurements. **: p < 0.01, ***: p < 0.001

We then investigated the effects of fruit juices on colony formation using human colon cancer LIM1215 cells. As shown in Fig. 1, Kakadu plum juice and pomegranate juice dose-dependently inhibited colony formation.

**Fig. 2:**
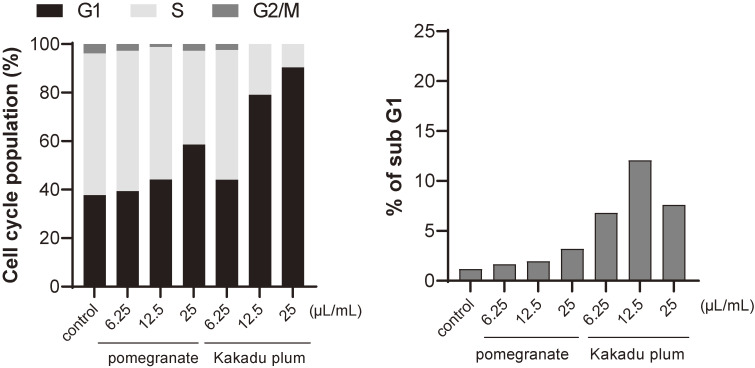
Effects of Kakadu plum juice and pomegranate juice on the cell cycle. LIM1215 cells were inoculated (1 × 10^5^ cells in 2 mL medium/well) and incubated in fresh medium with Kakadu plum juice or pomegranate juice at the indicated volumes for 24 hours after the synchronization of the cell cycle by serum-free medium for 2 days. The cell cycle was analyzed by flow cytometry. Each value is an average of two samples.

We performed a cell cycle analysis by flow cytometry of cells treated with several doses of Kakadu plum juice and pomegranate juice, which exerted potent inhibitory effects on colony formation. As shown in Fig. 2, Kakadu plum juice and pomegranate juice induced G0/G1 arrest, whereas the sub-G1 population was slightly increased by these juices.

### RB reactivation ability of two juices and their components

**Fig. 3:**
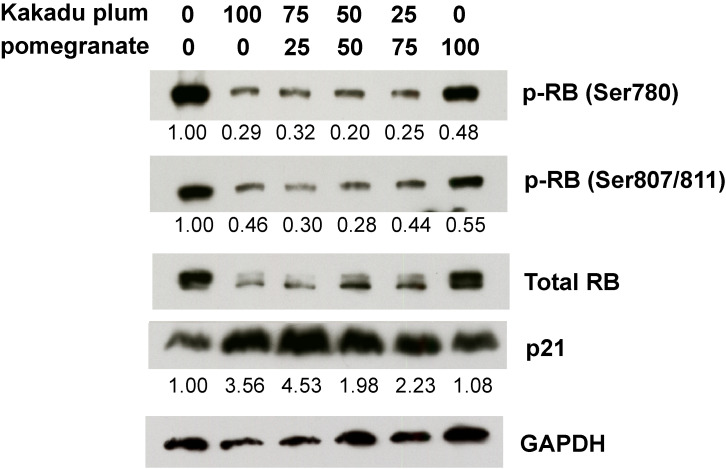
Western blot analysis of LIM1215 cells treated with juices. Mixed juice samples were prepared using the 2 juices at each ratio indicated. Each sample (12.5 µL) was added to LIM1215 cells at 2 mL medium/well and incubated for 72 hours. The expression levels of phosphorylated RB, total RB, and p21 were measured by Western blotting. GAPDH was used as a loading control. A semi-quantitative analysis of the intensities of target bands was performed after normalization based on GAPDH levels.

**Fig. 4:**
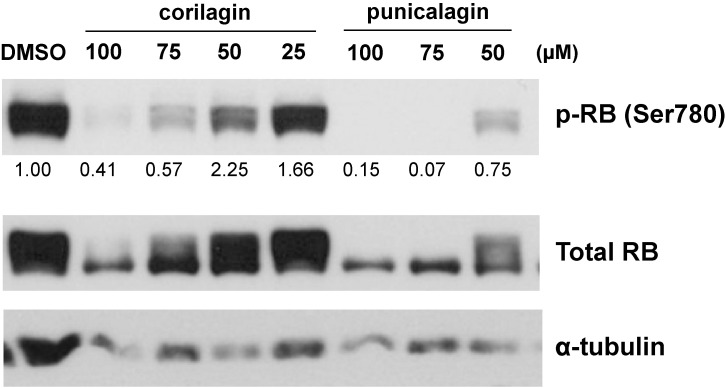
RB phosphorylation in LIM1215 cells treated with juice components. LIM1215 cells were treated with corilagin or punicalagin at the indicated concentrations for 72 hours. The expression levels of phosphorylated RB (Ser780) and total RB were evaluated by Western blotting. A semi-quantitative analysis of the intensities of target bands was performed after normalization based on α-tubulin levels.

To elucidate the mechanisms responsible for cell cycle arrest by the juice treatment, we performed Western blotting of the phosphorylation status of the RB protein, which is a key molecule in cell cycle control and cell growth. The phosphorylation of the RB protein was reduced 72 hours after the treatment with the mixture of Kakadu plum juice and pomegranate juice or each juice alone (Fig. 3). The reduction observed in the phosphorylation of the RB protein was greater with Kakadu plum juice than with pomegranate juice. The cyclin-dependent kinase inhibitor p21 was increased by the treatment with each juice and the mixed juice after 72 hours (Fig. 3). We speculated that these juices up-regulated the expression of p21 and converted the RB protein to its active form, resulting in G1 arrest. We additionally examined the effects of representative components of the juices on the activation of the RB protein. As shown in Fig. 4, corilagin and punicalagin, which are present in Kakadu plum juice and pomegranate juice [40–42], reduced phosphorylated RB protein levels after 72 hours, raising a possibility that these components might be candidates for the effects of the juices.

### Anti-inflammatory effects of juices

**Fig. 5:**
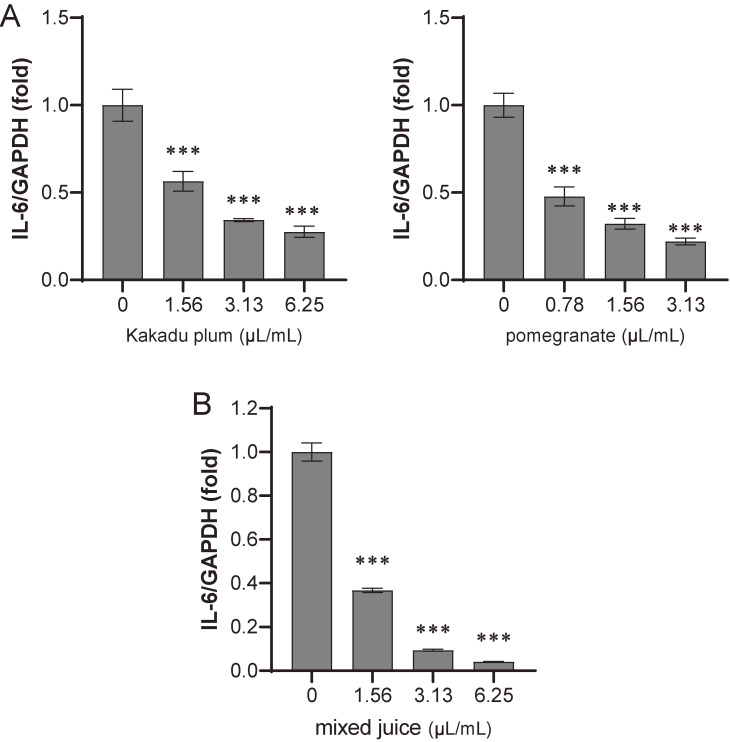
Kakadu plum and pomegranate juices inhibit TNF-α-induced IL-6 expression. LIM1215 cells were cultured with each sample for 1 hour. Cells were then treated with 10 ng/mL TNF-α for 1 hour. The expression level of IL-6 mRNA was measured by quantitative RT-PCR. The expression level of GAPDH was used for normalization. Each value is shown as a relative value against the control. A. Kakadu plum juice or pomegranate juice at the indicated doses. B. Mixture of 50% of each juice at the indicated doses. Data represent the means ± SD of three measurements. ***: p < 0.001

The anti-inflammatory effects of Kakadu plum juice and pomegranate juice were examined. Inflammatory events were evoked by TNF-α in human colon cancer LIM1215 cells, and induced IL-6 mRNA levels were evaluated. The quantity of IL-6 mRNA was normalized by GAPDH mRNA. As shown in Fig. 5A, both juices dose-dependently suppressed IL-6 mRNA expression, which is consistent with previous findings [43, 44].

Based on these results, the anti-inflammatory effects of the mixed juice were evaluated. As shown in Fig. 5B, the mixed juice also inhibited the induction of IL-6 in a dose-dependent manner.

### Antioxidant activities of juices

**Fig. 6:**
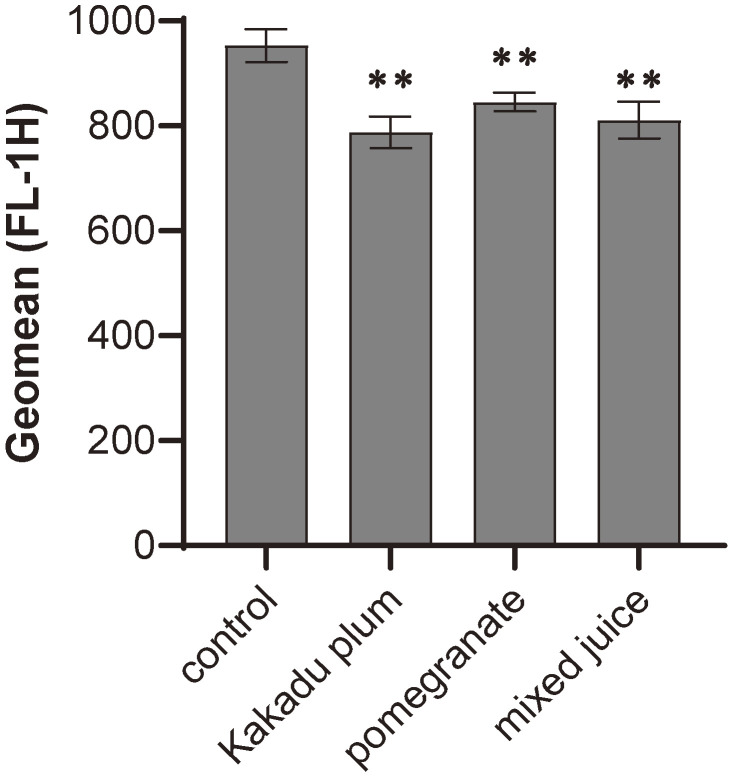
Kakadu plum and pomegranate juices inhibit the induction of reactive oxygen species by hydrogen peroxide. LIM1215 cells were cultured in medium containing 6.25 µL/mL of each juice for 24 hours. To induce reactive oxygen species, cells were treated with 500 µM H_2_O_2_ for the last 1 hour. Intracellular reactive oxygen species levels were measured using FACSCalibur with 10 µM CM-H_2_DCFDA as an indicator. Data were analyzed using Cell Quest software (n = 3). **: p < 0.01

ROS are harmful for living organisms because they cause genetic mutations and increase the risk of carcinogenesis. Kakadu plum and pomegranate have already been reported to possess antioxidant properties [43, 45]. Therefore, we herein investigated the antioxidant effects of these two fruit juices and the mixed juice. Each juice was added to LIM1215 cells at 6.25 µL/mL of the medium and incubated for 24 hours. In the last hour, 500 µM H_2_O_2_ was added to induce ROS production. The antioxidant capacity of each juice was examined by measuring intracellular ROS using flow cytometry with CM-H_2_DCFDA as a ROS indicator. The results obtained showed that Kakadu plum juice and pomegranate juice alone significantly scavenged ROS, while the mixed juice also significantly reduced ROS levels to the same extent (Fig. 6).

### Effects of *L. pentosus* S-PT84 on TRAIL expression in humans

**Fig. 7:**
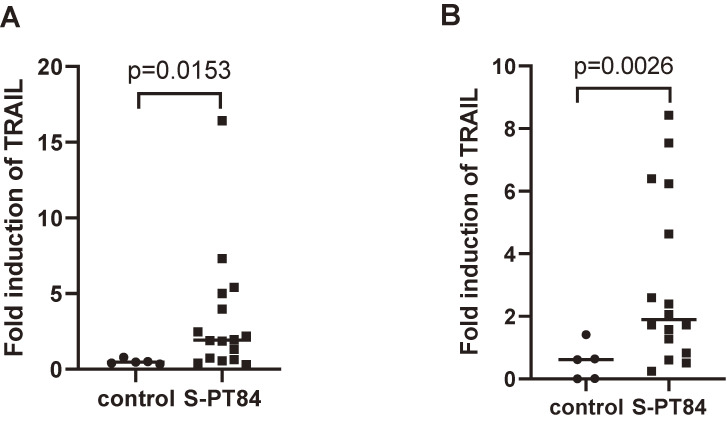
Induction of TRAIL protein levels in PBMC from healthy volunteers by the intake of S-PT84. Blood samples were collected from healthy volunteers who took placebo or S-PT84 tablets. Sampling was performed 1 week before the intake of tablets, 4 weeks after the start of the once-daily intake, and 4 weeks after the cessation of intake. A. Fold changes in TRAIL expression in the S-PT84 group (n = 16) and control group (n = 5) from before intake to 4 weeks after the start of the once-daily intake. B. Fold changes in TRAIL expression in the S-PT84 group (n = 16) and control group (n = 5) from before intake to 4 weeks after the cessation of intake. The quantity of TRAIL was normalized by GAPDH.

We investigated the effects of *L. pentosus* S-PT84 ingestion on TRAIL expression in healthy volunteers. Gender, age, and smoking history in both groups are listed in Additional file 1. Each volunteer took a placebo tablet (starch tablet) (n = 5) or S-PT84 tablet (n = 16) (0.5 billion of S-PT84 (n = 5), 1.5 billion of S-PT84 (n = 6) or 4.5 billion of S-PT84 (n = 5)) for 4 weeks. PBMC were isolated from blood samples. As shown in Fig. 7, the mean value of TRAIL/GAPDH protein expression was significantly higher in the S-PT84 group (n = 16) than in the control group (n = 5) (p = 0.0153) 4 weeks after the start of the once-daily intake of S-PT84 or placebo tablets (Fig. 7A), and remained elevated for the next 4 weeks (p = 0.0026) (Fig. 7B). In the comparison between a control group and each S-PT84 group, the number of individuals in each group was small, and the difference was not statistically significant enough, while the same tendency was observed by Welch’s t-test (Additional file 2).

### Preventive effects of juices and lactic acid bacteria against rat AOM-induced colon ACF

We then investigated the preventive effects of Kakadu plum juice with or without pomegranate juice and lactic acid bacteria using the rat AOM-induced colon ACF model.

Table 1 shows the preventive effects of juices and lactic acid bacteria in two independent experiments. The images presented in Table 1 show typical ACF with each number of ACs. In the first experiment, the number of ACF and the total number of ACs were significantly reduced by 22 and 19%, respectively, when 1.5 mL/day of Kakadu plum juice was orally administered for 5 days a week for 4 weeks.

**Table 1:**
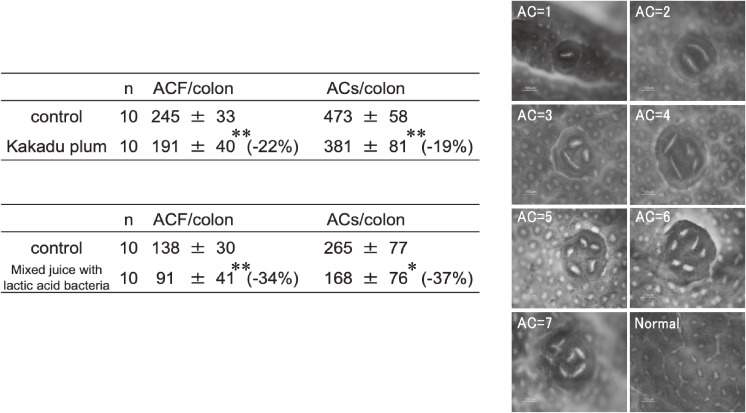
Inhibitory effects of each sample on the formation of ACF with ACs.

Mixed juice consisted of 50% Kakadu plum juice and 50% pomegranate juice. Images show typical ACF with the multiplicity of ACs on the colon mucosa under an optical microscope (magnification × 20). *: p < 0.05, **: p < 0.01

In the second experiment, the number of ACF and the total number of ACs were significantly reduced by 34 and 37%, respectively, when the same volume of a mixed juice of 50% Kakadu plum juice and 50% pomegranate juice with 0.01 g of dry powdered *L. pentosus* S-PT84 (5 × 10^10^ CFU/1 g of dry powder) was orally administered for 5 days a week for 4 weeks.

No adverse effects, such as diarrhea, body weight loss, or physiological changes in appearance, were observed in the three experiments (data are not shown).

## Discussion

Due to very rapid and easy screening, germ-line mutations that cause a predisposition to malignant tumors are now readily detected. Nevertheless, individualized agents to prevent carcinogenesis are not currently available. Mutations in tumor-suppressor genes are used to assess individual risks of carcinogenesis. For example, when the representative tumor-suppressor gene p53 is mutated in germ-line cells of Li-Fraumeni syndrome with a number of juvenile malignancies, the RB protein is phosphorylated and inactivated [8]. When the other representative tumor-suppressor gene p16 is mutated in germ-line cells of hereditary melanoma family, the RB protein is also phosphorylated and inactivated [8]. When another tumor-suppressor gene BRCA1 is mutated in the germ-line cells of the hereditary breast and ovarian cancer family, the RB protein is also phosphorylated and inactivated. Therefore, RB-reactivating juice may be beneficial for healthy individuals with carcinogenic variants of germ-line tumor-suppressor genes causing a variable predisposition to malignancies, and, thus, may have potential as ‘preemptive medicine’.

Regarding sporadic malignancies, the inactivation of the RB protein is also essential because it is inactivated in the majority of human malignant tumors due to the activation of oncogenes and inactivation of many tumor-suppressor genes [8]. Therefore, RB-reactivating juice may also be useful for the prevention of many sporadic malignancies.

We extensively searched for fruit juices or drinks exhibiting RB-reactivating activity and finally identified Kakadu plum juice and pomegranate juice with the activity. It is important to note that we had already identified many cancer-preventive or growth-inhibitory food factors exhibiting RB-reactivating activities [11–32]; however, these two juices were the first natural fruit juices to show this activity.

It is of great interest that the RB protein has also been reported to inhibit atherosclerotic abnormalities, myocardial ischemia, inflammation, neural disorders, such as Alzheimer’s or Parkinson’s diseases, amyotrophic lateral sclerosis, and stroke [46–59] in addition to the suppression of carcinogenesis. Therefore, RB reactivators may prevent atherosclerotic, inflammatory, and neural diseases as well as malignancies.

Stephen Friend, who cloned the RB gene [7], suggested the existence of a ‘Hero gene’ with the potential to prevent hereditary diseases (https://www.ted.com/talks/stephen_friend_the_hunt_for_unexpected_genetic_heroes) [60]. We herein propose that RB could be a novel candidate for the typical ‘Hero gene’ that might prevent various diseases.

As stated, Kakadu plum is a traditional bush food of the Aboriginal people in Australia. A small amount of Kakadu plum has recently been exported as a juice or processed food. However, it is still a wild fruit that has not been cultivated in commercial plantations. Kakadu plum contains many beneficial components, such as corilagin, punicalagin, ellagic acid, and high levels of vitamin C [40]. As far as we searched, there are no reports as to the influence of Kakadu plum juice on human or animal, and we can not discuss the mechanisms of the effects of Kakadu plum juice in detail by citing other information.

Components of pomegranate include punicalagin [41], corilagin [42], vitamin C, gallic acid, rutin, and ellagic acid [61]. Pomegranate is considered to be very promising for the prevention of variable malignancies [62]. Two phase II studies on pomegranate juice or extract for men with elevated prostate-specific antigen (PSA) levels following initial therapy for prostate cancer showed that pomegranate significantly increased the mean PSA doubling time [62–64]. Therefore, it is of great interest that pomegranate juice activated the RB protein in the present study.

The second important factor for the prevention of malignancies is anti-inflammatory [65] and antioxidant [66] effects. In the present study, Kakadu plum juice and pomegranate juice significantly exerted these effects. These results suggest that Kakadu plum juice and pomegranate juice have potential in the prevention of variable malignant tumors.

Anti-tumor immunity is also essential for cancer prevention. NK activity is considered to be important for the prevention of cancer [67]. We and others have previously shown that lactic acid bacteria stimulated NK activity [38, 68]. We also demonstrated that *L. plantarum* strains enhanced the expression of the cancer-preventive TRAIL protein as well as NK activity against cancer cells [38]. The present study is the first to show that the expression of the TRAIL protein was stimulated by lactic acid bacteria in humans. TRAIL is important for the prevention of cancer [37], and lactic acid bacteria may be useful for cancer prevention by enhancing NK activity and up-regulating the expression of TRAIL. Previous studies demonstrated that lactic acid bacteria reduced the risk of colorectal tumors [69] and breast cancer in humans [70].

Therefore, we herein examined the effects of a mixed juice of Kakadu plum and pomegranate with lactic acid bacteria using rat AOM-induced colon ACF. The mixed juice with lactic acid bacteria significantly reduced the number of ACF and total number of ACs by 34 and 37%, respectively. We speculate that these remarkable effects may be attributed to the combination of RB-reactivating, anti-inflammatory, antioxidant, and anti-tumor immunity effects.

Collectively, the present results indicate that the reactivation of RB could be a very essential factor for the prevention of cancer as well as cancer therapy [8].

## Conclusions

RB has been extensively examined and is known to inhibit carcinogenesis in most malignancies. Furthermore, it is an essential molecule in the treatment or prevention of malignancies. Nevertheless, natural juices or drinks have not been shown to activate the functions of the RB protein. To the best of our knowledge, this is the first study to demonstrate that natural juices, not their constituents or extracts, activated the functions of RB. It is important to note that the oral administration of Kakadu plum and pomegranate juices, which activate RB functions and exert anti-inflammatory and antioxidant effects, in combination with lactic acid bacteria, which stimulate anti-tumor immunity, significantly inhibited ACF and ACs in the rat AOM-induced colon ACF model.

We herein propose the potential of a multi-functional drink including ‘RB-reactivating functions’ as a novel, effective, and safe cancer-preventive strategy.

## Abbreviations

RB: retinoblastoma gene
TRAIL: tumor necrosis factor-related apoptosis-inducing ligand
ACF: aberrant crypt foci
ACs: aberrant crypts
*L.*: *Lactiplantibacillus*
COX-2: cyclooxygenase-2
PBMC: peripheral blood mononuclear cells
NK: natural killer
GAPDH: glyceraldehyde-3-phosphate dehydrogenase
HRP: horseradish peroxidase
DMEM: Dulbecco’s modified Eagle’s medium
AOM: azoxymethane
TNF-α: tumor necrosis factor-α
IL-6: interleukin 6
SDS-PAGE: sodium dodecyl sulfate-polyacrylamide gel electrophoresis

## Supplementary information

**Additional file 1:** Basic information of a control group and S-PT84 groups in the human trial. Prior to the study, participants in the study were interviewed for information on gender, age and smoking history. Based on this result, grouping was performed. An upper panel is the table of the information of a control group and an S-PT84 group, and a lower panel is the table of the information of a control group and S-PT84 groups (each 0.5 billion/day, 1.5 billion/day or 4.5 billion/day of an S-PT84 group).

**Additional file 2:** Induction of TRAIL protein levels in PBMC from healthy volunteers by the intake of S-PT84. Blood samples were collected from healthy volunteers who took placebo or S-PT84 tablets. Sampling was performed 1 week before the intake of tablets, 4 weeks after the start of the once-daily intake, and 4 weeks after the cessation of the intake. Fold changes in TRAIL expression at 1 week before the intake to 4 weeks later (upper row) or 8 weeks later (lower row) after the intake were shown in each group of S-PT84 (0.5 billion group: n = 5, 1.5 billion group: n = 6, 4.5 billion group: n = 5) and a control group (n = 5).

## Declarations

### Availability of data and materials

The data and materials during the current study are available from the corresponding author on reasonable request.
