# Supplementary material for: Discovery of cancer-preventive juices reactivating RB functions
Source: Environ Health Prev Med. 2023 Sep 22;28:54. doi: 10.1265/ehpm.23-00160 (PMC10519803; doi:10.1265/ehpm.23-00160)
Supplement: Supplementary file 1 — Additional file 1: Basic information of a control group and S-PT84 groups in the human trial. Prior to the study, participants in the study were interviewed for information on gender, age and smoking history. Based on this result, grouping was performed. An upper panel is the table of the information of a control group and an S-PT84 group, and a lower panel is the table of the information of a control group and S-PT84 groups (each 0.5 billion/day, 1.5 billion/day or 4.5 billion/day of an S-PT84 group). [file ehpm-28-054-s001.pptx]

## Slide 1
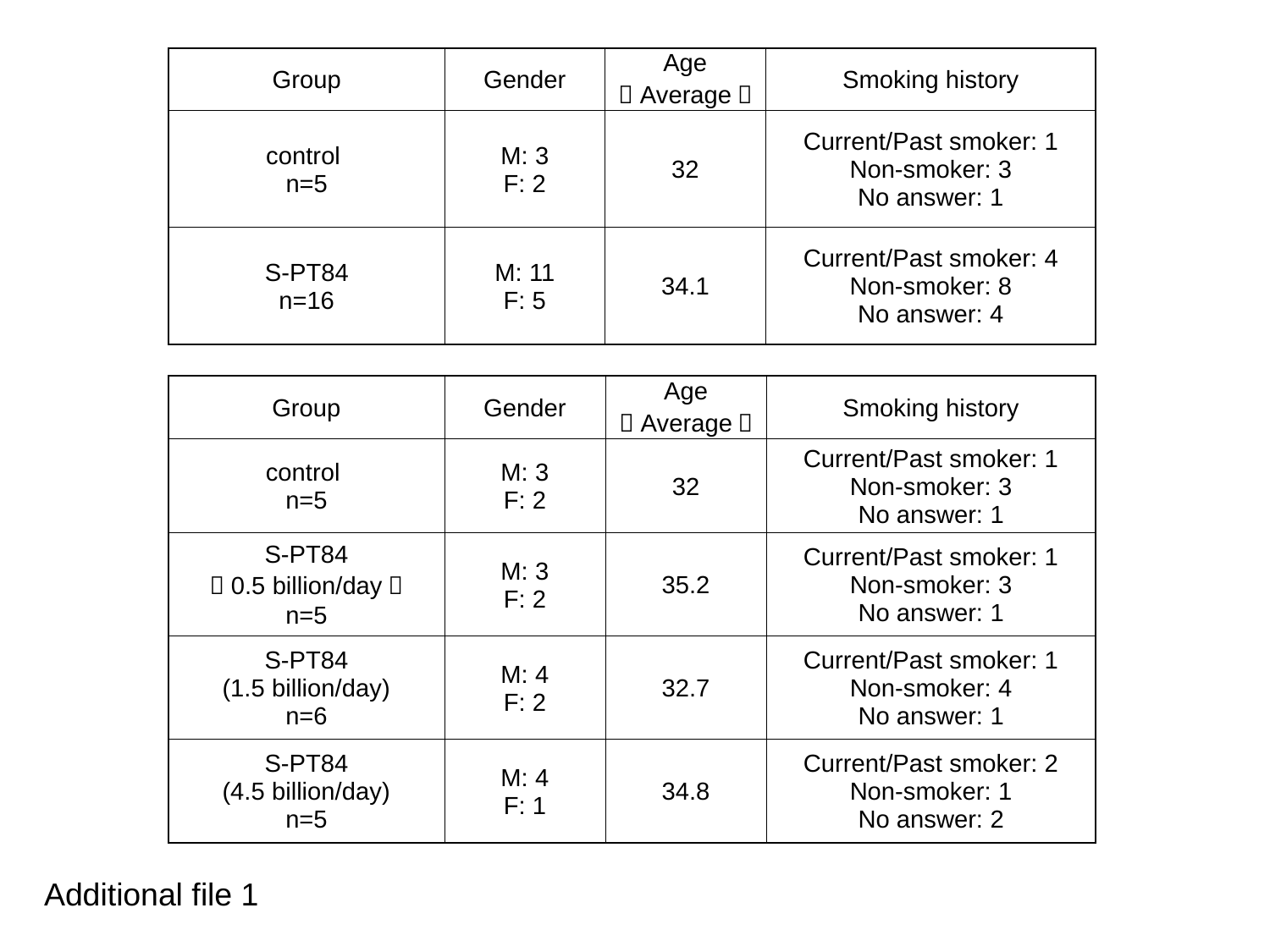

| Group | Gender | Age （Average） | Smoking history |
| --- | --- | --- | --- |
| control n=5 | M: 3 F: 2 | 32 | Current/Past smoker: 1 Non-smoker: 3 No answer: 1 |
| S-PT84 n=16 | M: 11 F: 5 | 34.1 | Current/Past smoker: 4 Non-smoker: 8 No answer: 4 |
| Group | Gender | Age （Average） | Smoking history |
| --- | --- | --- | --- |
| control n=5 | M: 3 F: 2 | 32 | Current/Past smoker: 1 Non-smoker: 3 No answer: 1 |
| S-PT84 （0.5 billion/day） n=5 | M: 3 F: 2 | 35.2 | Current/Past smoker: 1 Non-smoker: 3 No answer: 1 |
| S-PT84 (1.5 billion/day) n=6 | M: 4 F: 2 | 32.7 | Current/Past smoker: 1 Non-smoker: 4 No answer: 1 |
| S-PT84 (4.5 billion/day) n=5 | M: 4 F: 1 | 34.8 | Current/Past smoker: 2 Non-smoker: 1 No answer: 2 |
Additional file 1
