# Supplementary figures and images for: Discovery of cancer-preventive juices reactivating RB functions
Source: Environ Health Prev Med. 2023 Sep 22;28:54. doi: 10.1265/ehpm.23-00160 (PMC10519803; doi:10.1265/ehpm.23-00160)

## Slide 1
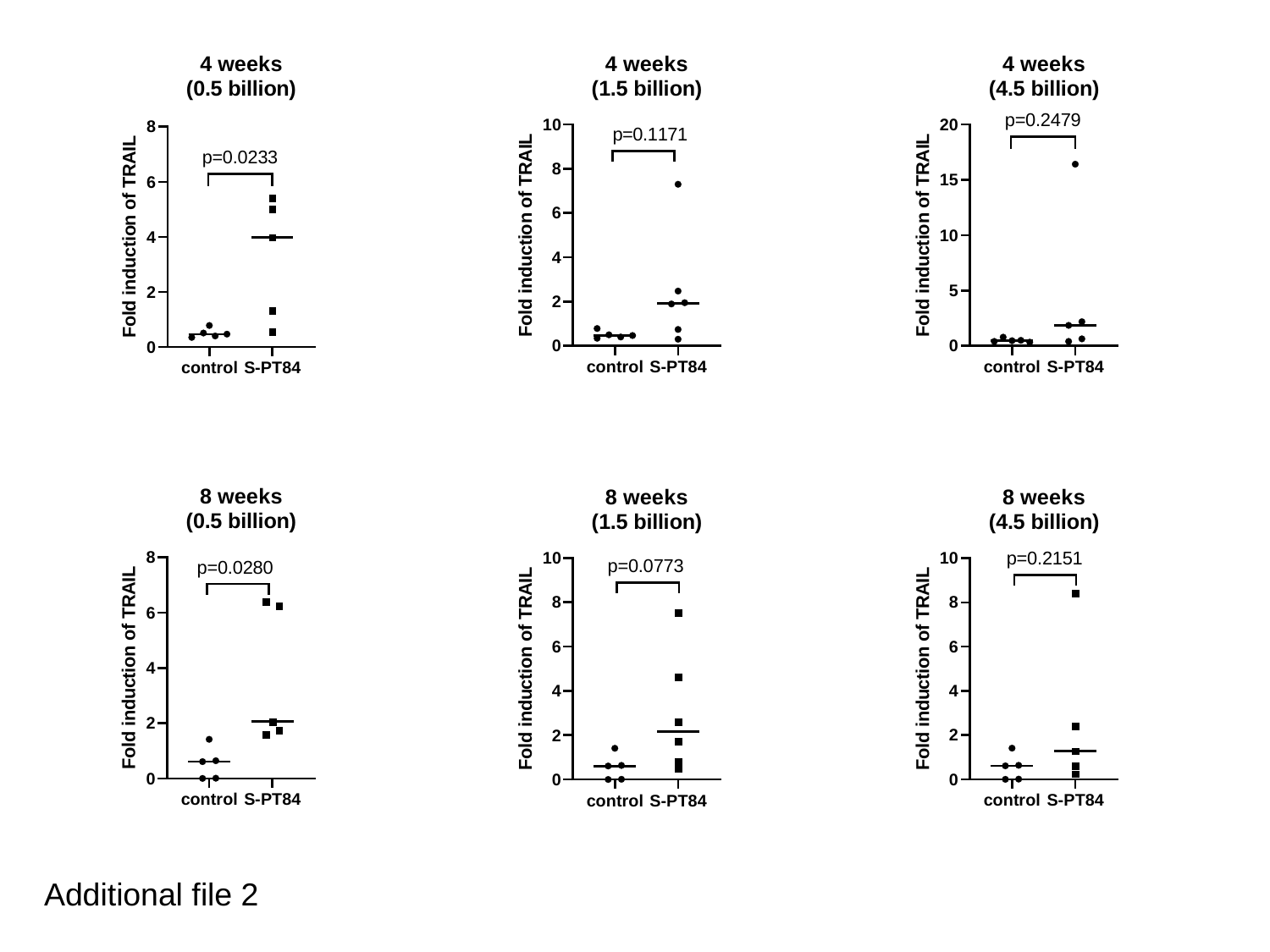

Additional file 2

Supplement: Supplementary file 2 — Additional file 2: Induction of TRAIL protein levels in PBMC from healthy volunteers by the intake of S-PT84. Blood samples were collected from healthy volunteers who took placebo or S-PT84 tablets. Sampling was performed 1 week before the intake of tablets, 4 weeks after the start of the once-daily intake, and 4 weeks after the cessation of the intake. Fold changes in TRAIL expression at 1 week before the intake to 4 weeks later (upper row) or 8 weeks later (lower row) after the intake were shown in each group of S-PT84 (0.5 billion group: n = 5, 1.5 billion group: n = 6, 4.5 billion group: n = 5) and a control group (n = 5). [file ehpm-28-054-s002.pptx]
